# The N-Terminal, Polybasic Region Is Critical for Prion Protein Neuroprotective Activity

**DOI:** 10.1371/journal.pone.0025675

**Published:** 2011-09-29

**Authors:** Jessie A. Turnbaugh, Laura Westergard, Ursula Unterberger, Emiliano Biasini, David A. Harris

**Affiliations:** 1 Department of Biochemistry, Boston University School of Medicine, Boston, Massachusetts, United States of America; 2 Department of Cell Biology and Physiology Washington University School of Medicine St. Louis, St. Louis, Missouri, United States of America; University of Melbourne, Australia

## Abstract

Several lines of evidence suggest that the normal form of the prion protein, PrP^C^, exerts a neuroprotective activity against cellular stress or toxicity. One of the clearest examples of such activity is the ability of wild-type PrP^C^ to suppress the spontaneous neurodegenerative phenotype of transgenic mice expressing a deleted form of PrP (Δ32–134, called F35). To define domains of PrP involved in its neuroprotective activity, we have analyzed the ability of several deletion mutants of PrP (Δ23–31, Δ23–111, and Δ23–134) to rescue the phenotype of Tg(F35) mice. Surprisingly, all of these mutants displayed greatly diminished rescue activity, although Δ23–31 PrP partially suppressed neuronal loss when expressed at very high levels. Our results pinpoint the N-terminal, polybasic domain as a critical determinant of PrP^C^ neuroprotective activity, and suggest that identification of molecules interacting with this region will provide important clues regarding the normal function of the protein. Small molecule ligands targeting this region may also represent useful therapeutic agents for treatment of prion diseases.

## Introduction

Prion diseases are invariably fatal neurodegenerative disorders resulting from the conversion of the normally α-helical cellular prion protein (PrP^C^) into a misfolded β-sheet rich conformer called PrP^Sc^. While much research has focused on characterizing PrP^Sc^ as an infectious agent, little progress has been made in defining the normal function of PrP^C^. Mice deleted for endogenous PrP are relatively normal, with no gross anatomical or developmental defects, providing few clues for understanding the physiological role of this protein [1], [2].

Several studies attempting to characterize PrP^C^ function demonstrated that the protein may have a role in neuroprotection. For example, overexpression of PrP^C^ has been shown to protect cells against a variety of apoptotic stimuli, including Bax overexpression [3], [4], oxidative stress [5], [6], and serum-deprivation [7], [8]. However, in almost all cases PrP^C^ expression provided only a modest neuroprotective effect, making these cell assays difficult to reproduce [9] and calling into question their physiological relevance.

Perhaps one of the most dramatic examples of PrP-dependent neuroprotection has been observed in mice expressing mutant forms of the protein. Transgenic expression of PrP molecules deleted for residues 32–121, 32–134, 105–125 or 94–134 leads to a spontaneous neurodegenerative phenotype [10], [11], [12], as does ectopic expression of Doppel, a PrP paralog structurally homologous to the C-terminal half of PrP [13], [14], [15], [16].

Intriguingly, co-expression of wild type (WT) PrP counteracts the neurodegenerative effect of each of these PrP mutants and Doppel, providing a way to test PrP neuroprotective activity *in vivo*. For example, PrP molecules deleted for most (Δ32–80) or all (Δ32–93) of the octapeptide repeats rescued mice expressing Δ32–134 PrP [referred to as Tg(F35)] as efficiently as WT PrP, indicating that this region is not essential for neuroprotection [10], [17]. Conversely, PrP carrying a deletion of 23–88 had an impaired ability to rescue from Doppel, despite being expressed at higher levels than WT PrP [18]. Collectively, these results suggest that the N-terminus of PrP, particularly residues 23–31, is critical for PrP rescuing activity. These residues (^23^KKRPKPGGW^31^) are highly conserved across mammalian species and have several known functions, including regulating PrP endocytosis [19], [20], [21], binding to glycosaminoglyans (GAGs) [22], [23], [24] and the ability to act as a protein transduction domain [25].

In order to directly address the role of residues 23–31 in the neuroprotective activity of PrP, we have compared the ability of several specific N-terminal deletion mutants, including Δ23–31, Δ23–111, or Δ23–134 PrP, to reverse F35-induced toxicity in Tg mice. We found that each of these molecules showed greatly impaired rescuing activity, despite considerably higher expression levels compared to WT PrP. These results demonstrate that residues 23–31 are crucial for the neuroprotective function of PrP.

## Materials and Methods

### Ethics Statement

This study was carried out in strict accordance with the recommendations in the Guide for the Care and Use of Laboratory Animals of the National Institutes of Health. The protocol was approved by the Boston University Institutional Animal Care and Use Committee (Permit Number: AN-14997).

### Construction of transgenic mice

Bridge PCR amplification was used to generate Δ23–31 PrP (containing a 3F4 epitope tag) using in the yeast p426GPD vector [26]. Δ23–31 PrP was subcloned into the mammalian pCNDA3.1 (+) Hygro vector (Invitrogen, Carlsbad, CA) under the control of the CMV promoter. To create a non 3F4-tagged version of this plasmid, the C-terminal (3F4-containing) fragment of the plasmid described above was released by digestion with restriction enzymes AgeI and XbaI and the corresponding portion of the untagged cDNA was ligated to the Δ23–31 vector. Using this non-3F4-tagged Δ23–31 construct, the following primers were used to PCR-amplify PrP: FWD (5′ – TATATACTC GAGGCCGCCACCATGGCGAACCTTGGCTACTGG – 3′) and REV (5′ – CTCGAGCTT GTCATCGTCGTCCTTGTAGTCTCATTATCCCACGATCAGGAAGATGAG – 3′).

A cDNA encoding murine C1 (Δ23–111) was generated by PCR amplification. The following primers were used: FWD (5′ – TCCGA AAGCTTCTCGAGGCCGCCACCA TGGCGAACCTTGGCTACTGGCTGCTGGCCCTCTTTGTGACTATGTGGACTGATGTCGGCCTCTGCAGGCCCATGATCCATTTTGGC – 3′) and REV (5′ – CGGACTCTAGACT CGAGTCATCATCCCACGATCAGGAAGAT – 3′). The resulting PCR product was digested with HindIII and XbaI and cloned into pcDNA 3.1 (+) Hygro.

To create both Tg (Δ23–31) and Tg (Δ23–111) mice, the corresponding sequences were released from the pcDNA3.1 (+) Hygro plasmid by digestion with XhoI and ligated into the Xho I site of MoPrP.Xho [27] under the control of the mouse half-genomic PrP promoter. The resulting colonies were checked for the presence of the insert using PCR primers P1 and P4 [28], and then sequenced to confirm the correct sequence and orientation. The transgene was released from the recombinant plasmid by NotI digestion, purified with a GFX column (GE), and injected into the pronuclei of fertilized eggs from C57BL6/J×CBA hybrid mice. Tg (Δ23–31) founders were bred to *Prn-p^−/−^* mice on the C56BL6/J background (EMMA), and Tg(Δ23–111) founders were bred initially to Tga20^+/+^ mice on a C57BL6/CBA/129 background (EMMA), and were then back-crossed to *Prn-p^−/−^* mice on the C56BL6/J background.

Generation of Tg(Δ23–134) mice has been described elsewhere [29]. Mice expressing Δ23–31, Δ23–111, or Δ23–134 on the *Prn-p^−/−^* background were mated to F35^+/0^ *Prn-p^+/−^* mice to generate the genotypes used in this study. All transgenes were hemizygous.

### Genotyping of transgenic mice

Mice were genotyped by PCR analysis of tail DNA prepared using the Puregene DNA Isolation Kit (Gentra Systems, Minneapolis, MN). The *Prn-p* allele was detected with primers E2 (referred to as P2 in [28]) and E4 [12]. Primers E2 and K4 (5′ – GTGAG ATGACAGGAGATCCTGCC – 3′) recognized the PrP knockout allele. Primer pair 890 (5′ – CTCGAGGCCGCCACCATG – 3′) and P4 [28] recognized Δ23–31, Δ23–111, and Δ23–134 PrP transgenes, and primers 913 (5′ – AAGCGGCCAAAGCCTGGAGGGTGG – 3′) and P4 recognized the F35 transgene.

### Statistical Analysis

Animals were sacrificed when terminally ill. The age of each mouse at the time of sacrifice was used to compare the lifespans of mice of the indicated genotypes using the Kruskal-Wallis test with Dunn's secondary testing. All statistical analyses were performed using the GraphPad Prism 5 program.

### Immunofluorescence and PIPLC treatment

BHK cells grown in PDL-coated 8 well chamber slides (BD Biosciences) were transiently transfected w/0.25 µg DNA and 0.75 µg Lipofetamine2000 (Invitrogen) per well. At 24 hours post-transfection, cells were washed with PBS, fixed in 4% paraformaldehyde for 10 minutes, permeabilized with 0.2% Triton X-100, and blocked in 2% goat serum in PBS. Cells were then stained with the following antibodies in blocking solution: 6D11 (R. Kascsak), 6H4 (Prionics), and Giantin (Covance), followed by incubation with fluorescently conjugated secondary antibodies (Molecular Probes), staining with DAPI, and visualization with a fluorescence microscope. For surface staining and PIPLC treatment, the same transfection protocol was followed except that plasmids encoding PrP and dsRedER were co-transfected (0.25 µg DNA each). At 24 hours post-transfection, cells were incubated in the absence or presence of PIPLC (Sigma) at 0.5 U/ml for 2 hours prior to surface staining with anti-PrP antibody 6D11 or 6H4, followed by incubation with a fluorescently conjugated secondary antibody (Molecular Probes) and staining with DAPI.

### Cerebellar graunule cell cultures

Cultures were performed as described previously [12]. After 4–5 days in culture, cells were fixed with 4% paraformaldehyde, surface stained with anti-PrP antibody 6H4, and then incubated with AlexaFlour 488-coupled goat anti-mouse IgG.

### Histology

Mouse brains were fixed in 4% paraformaldehyde before embedding in paraffin and cutting 4 µm sections. Paraffin sections were stained with hematoxylin and eosin as described previously [28], and were imaged with a Nikon TE-2000E inverted microscope.

### PNGaseF treatment and Western Blotting

Brain homogenates (10% w/v) were made by mechanically dissociating frozen brains in PBS using plastic pestles (South Jersey Precision Tool and Mold Inc., Vineland, NJ). Homogenates were then lysed in 0.5% NP-40/0.5% DOC, pH 7, and total protein levels were quantified with the BCA kit (Pierce, Rockford, IL). To de-glycosylate PrP, a 20 µg aliquot of total protein was treated with PNGase-F (N-glycosidase-F, New England Biolabs, Beverly, MA) according to the manufacturer's instructions. Samples were subjected to Western blotting and probed with anti-PrP antibody 6H4 (Prionics) followed by goat anti-mouse IgG (Pierce, Rockford, IL). Blots were developed with Millipore immobilon Western Chemiluminescent HRP substrate prior to imaging on a Biorad Chemidoc XRS system.

### Immunoprecipitation

Brain homogenates (10% w/v in PBS) were lysed in 0.5% CHAPS/0.5% NP-40 containing protease inhibitors (complete Mini EDTA-free, Roche), subjected to low-speed centrifugation to remove DNA and cellular debris, and total protein was quantitated using the BCA kit. Prior to immunoprecipitation, 30 µg of 6D11 antibody was coupled to 500 µl of anti-IgG Dynabeads (Dynal, Carlsbad, CA) in presence of 20 mM dimethyl pimelimidate dihydrochloride (Sigma), followed by washing and resuspending in PBS containing 0.1% BSA. Lysates were diluted to 0.5 mg/ml, pre-cleared with naked beads, and PrP was immunoprecipitated overnight with 50 µl of antibody-coupled Dynabeads, or with naked beads, washed, and collected with a magnet. Beads were re-suspended in 0.5% NP-40/0.5% DOC, pH 7, and digested with PNGase-F as described above. After digestion, samples were boiled in SDS-loading sample buffer prior to Western blotting.

## Results

### N-terminal PrP deletion mutants have a cellular localization pattern similar to WT PrP

**Figure 1:**
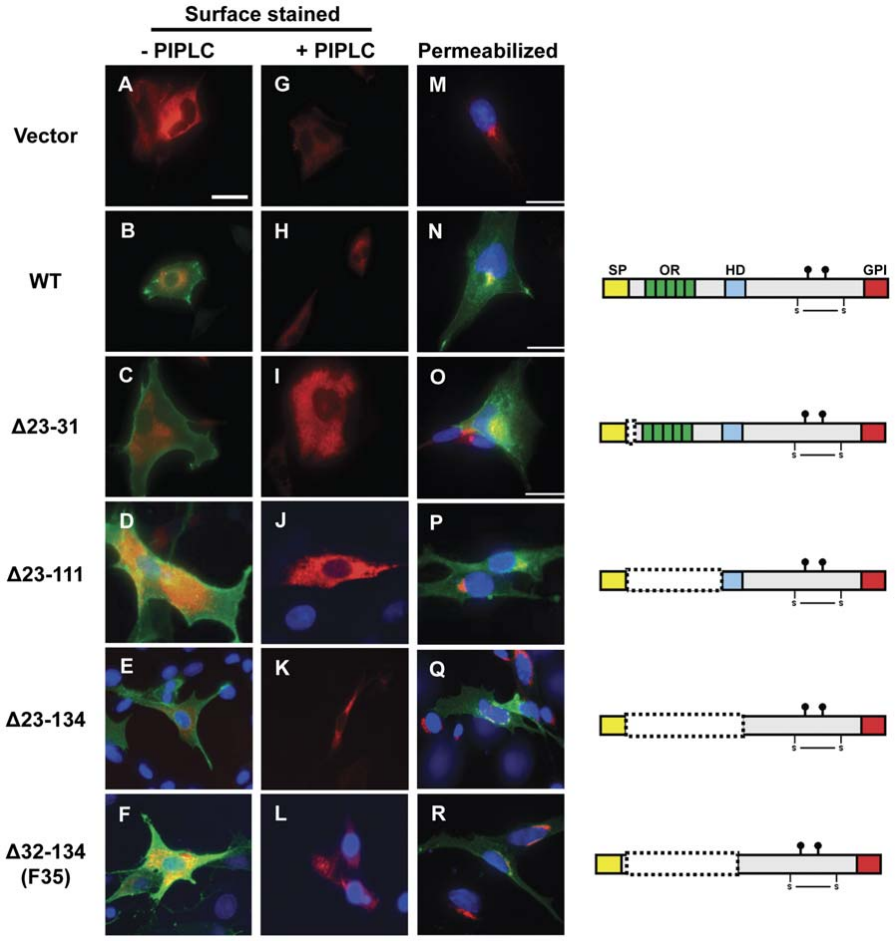
Δ23–31, Δ23–111, Δ23–134, and Δ32–134 (F35) PrP are GPI-anchored and have a cellular localization pattern similar to WT PrP. (**A–L**) The indicated constructs were transiently expressed in BHK cells along with dsRedER. Cells were incubated in the absence (A–F) or presence (G–L) of PIPLC, then surface stained for PrP (A–C, G–I: 6D11 or D–F, J–L: 6H4) on ice prior to incubating with secondary antibody (dsRedER signal in red, PrP in green). DAPI staining is shown in blue for panels D–F, J–L. Like WT PrP (H), the mutant PrP molecules are released from the plasma membrane by PIPLC treatment (I–L). (**M–R**) BHK cells transfected with the indicated constructs were permeabilized and stained with anti-PrP antibody [M–O: 6D11, P–R: 6H4 (green)], anti-giantin antibody (red), and DAPI (blue). Like WT PrP, each mutant is present both at the cell surface and intracellularly, where it colocalizes with the Golgi marker, giantin. [Scale bar in A (applicable to panels A–L, P–R) = 25 µm. Scale bar in M–O = 15 µm.].

Before examining the ability of N-terminal deletion mutants to rescue the toxicity of F35 PrP *in vivo*, we characterized the localization of these proteins in cultured cells. To demonstrate that Δ23–31, Δ23–111, and Δ23–134 are correctly delivered to the plasma membrane, BHK cells expressing either WT or mutant PrP were incubated with or without phosphatidylinositol-specific phospholipase C (PIPLC) then surface-stained with an anti-PrP antibody. We found that, like WT PrP, Δ23–31, Δ23–111, Δ23–134, and F35 PrPs were released by PIPLC treatment, demonstrating that they are all attached to the outer leaflet of the plasma membrane via a phospholipase-cleavable GPI anchor (Figure 1 A–L). Additionally, WT PrP and each of the N-terminal mutants co-localized with the Golgi marker, giantin, in permeabilized BHK cells, indicating that the proteins traffic through the Golgi on their way to the plasma membrane (Figure 1 M–R).

**Figure 2:**
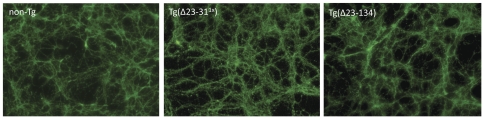
Cell surface staining of PrP in cerebellar neurons from non-Tg, Tg(Δ23–31^1×^), and Tg(Δ23–134) mice. Cerebellar granular neurons cultured from mice of the indicated genotype were stained for cell surface PrP (6H4, green). Like WT PrP from non-Tg mice, both Δ23–31 and Δ23–134 PrPs are present on the cell surface and along neurites.

To confirm that deletion of the N-terminal residues does not alter PrP localization in neurons, we also examined the localization of Δ23–31 and Δ23–134 PrP in cerebellar granule neurons cultured from the respective transgenic mice. Immunofluorescent staining of cell surface PrP showed that, like WT PrP, Δ23–31 and Δ23–134 PrPs are expressed on the plasma membrane of cell bodies as well as neurites (Figure 2).

### Deletions of the N-terminus of PrP compromise its rescuing ability

**Figure 3:**
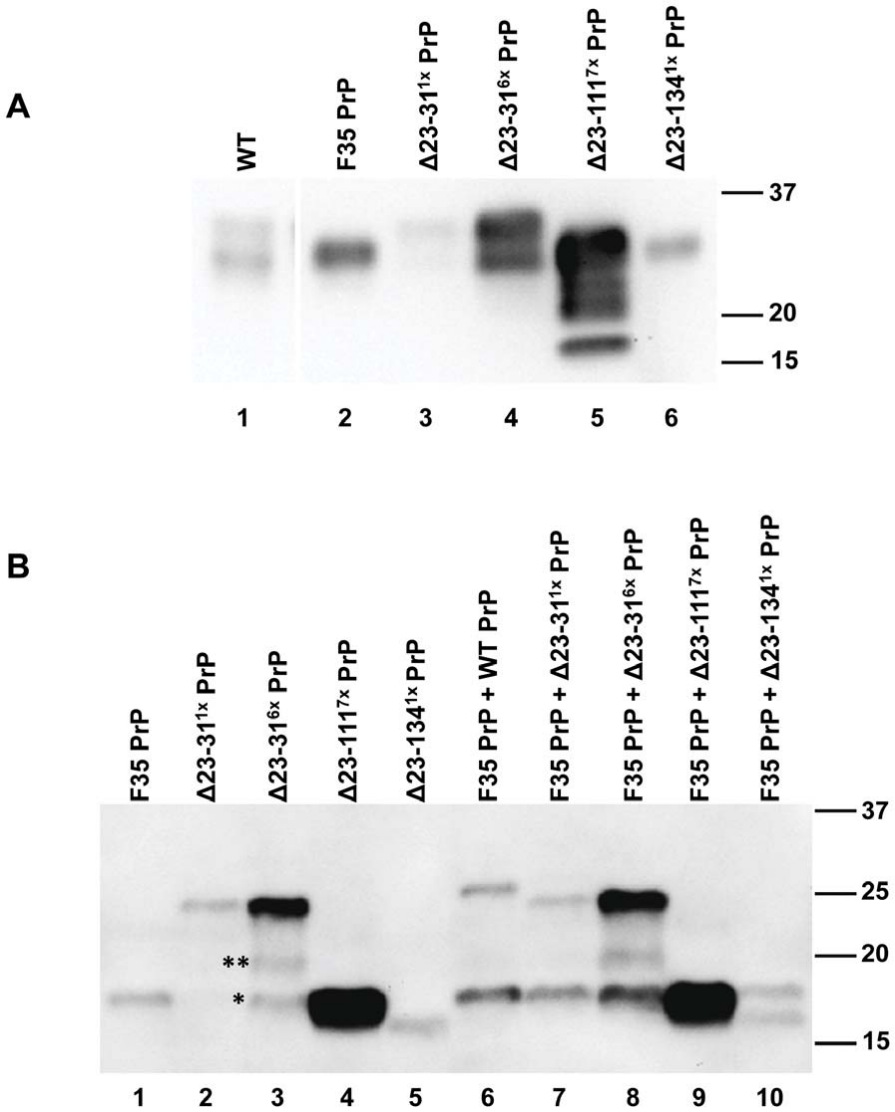
Expression of transgenes. (**A**) Brain lysates from a non-transgenic WT mouse (expressing 1× PrP), and from Tg mice expressing F35 PrP (2×), Δ23–31 PrP (1× and 6×), Δ23–111 PrP (7×), and Δ23–134 PrP (1×) were Western blotted and probed with anti-PrP antibody 6H4. (**B**) Lysates from the brains of 10 week old mice were treated with PNGase F to removed N-linked oligosaccharides. Digestion products were subjected to Western blotting using antibody 6H4 to detect PrP. Single and double asterisks mark the positions of the endogenous C1 and C2 cleavage fragments, respectively.

In order to define the role of the N-terminal region of PrP in neuroprotection, we compared the lifespan of mice co-expressing F35 PrP along with either WT PrP or three different, N-terminally deleted mutants (Δ23–31, Δ23–111, or Δ23–134). All transgenes were expressed under the control of the PrP half-genomic promoter on a *Prn-p^−/−^* background. Δ23–111 PrP corresponds to the major, physiologically occurring, C-terminal fragment of PrP, called C1. In this study, we utilized two lines of Tg(Δ23–31) mice with expression levels of 1× and 6× with respect to endogenous PrP, one line of Tg(Δ23–111) mice with an expression level of 7×, and one line of Tg(Δ23–134) mice with an expression level of 1× (Figure 3A, compare lanes 3–6 to lane 1). The Tg(F35) line expresses the mutant protein at 2× (Figure 3A, lane 2) [10]. As shown in Figure 3, each mutant migrated at the expected molecular weight and was glycosylated, with the di-glycosylated band appearing as the predominant form.

Tg(F35)/*Prn-p^+/−^* mice were crossed with Tg(Δ23–31^1×^), Tg(Δ23–31^6×^), Tg(Δ23–111^7×^), or Tg(Δ23–134^1×^), all on a *Prn-p* ^−/−^ background, to retrieve the doubly transgenic genotypes shown in Table 1. To assess relative expression levels of the mutant PrP molecules, we treated brain homogenates taken from mice at 10 weeks of age with PNGase F to removed N-linked oligosaccharides, followed by Western blotting (Figure 3B). These results demonstrated that levels of Δ23–31, Δ23–111, or Δ23–134 PrPs were not affected by co-expression of the F35 mutant, and conversely that the level of F35 PrP was not affected by co-expression of the other mutants (Figure 3B, lanes 6–10).

**Table 1:** N-terminally deleted forms of PrP are impaired in their ability to suppress the neurodegenerative phenotype of Tg(F35) mice.

Genotype	Expression level of rescue molecule	Age at death (days)
Tg(F35)/*Prn-p^−/−^*	0	88.1±8.1 (n = 30)
Tg(F35)/*Prn-p^+/−^*	0.5×	>365 (n = 12) **
Tg(F35/Δ23–31)/*Prn-p^−/−^*	1×	100.9±14.1 (n = 12)
Tg(F35/Δ23–31)/*Prn-p^−/−^*	6×	159.1±22.2 (n = 10) **
Tg(F35/Δ23–111)/*Prn-p^−/−^*	7×	97.8±10.2 (n = 10)
Tg(F35/Δ23–134)/*Prn-p^−/−^*	1×	126.4±14.2 (n = 8) *

The genotype, number of mice, age at death, and relative expression levels PrP are shown for each transgenic line. While 0.5× expression of WT PrP greatly prolongs the lifespan of Tg(F35) mice, the N-terminal mutants have only a modest effect on lifespan, even at elevated expression levels. Asterisks indicate statistically significant differences in age at death compared to Tg(F35)/*Prn-p* ^−/−^ mice (** p<0.001, * p<0.01 by Kruskal-Wallis with Dunn's secondary test).

Mice expressing Δ23–31, Δ23–111, or Δ23–134 PrP in the absence of F35 PrP showed no evidence of spontaneous disease and had normal lifespans (not shown).

**Figure 4:**
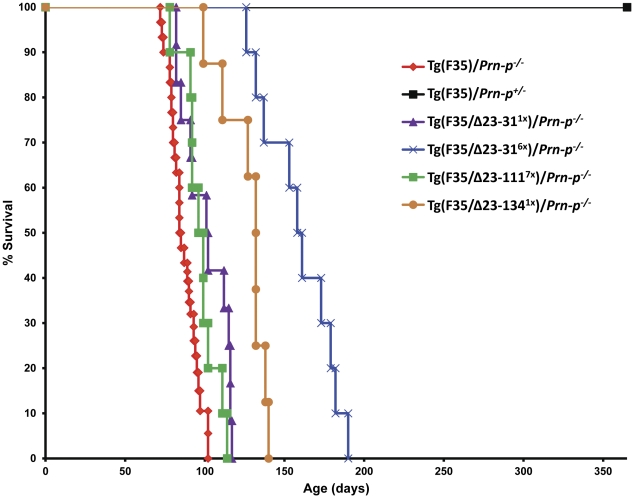
Survival of mice co-expressing N-terminal deletion mutants. Each point represents the percentage of animals alive at the indicated age. Statistical analyses are shown in Table 1.

As reported previously [10], co-expression of 0.5× WT PrP completely suppressed neurological signs of disease and extended the lifespan of F35 mice to more than 1 year (Table 1; Figure 4, black line). In contrast, co-expression of each of the three N-terminal deletion mutants did not delay the age of onset (data not shown) and failed to reverse the F35 clinical phenotype, with all mice displaying progressive tremor, ataxia, and hind limb paresis and eventually dying from neurological illness. Moreover, each of the mutant PrP molecules had a much weaker effect than WT PrP on extending the lifespan of the mice. For example, although 6× expression of Δ23–31 PrP produced a statistically significant lengthening of lifespan, 1× expression had no significant effect on survival (Table 1; Figure 4, blue and purple lines, respectively). The Δ23–111 mutant, which carries a larger deletion, provided no statistically significant rescue even when expressed at 7× (Table 1; Figure 4, green line). Surprisingly, Δ23–134 PrP showed a more substantial rescue than the other two mutants, despite the fact that it harbors the longest deletion and was expressed at only 1× (Table 1; Figure 4, orange line).

Collectively, these results indicate that each of the N-terminal deletion mutants is impaired compared to WT PrP in its ability to suppress neurological symptoms and death in Tg(F35) mice, even when expressed at supraphysiological levels. Moreover, deletion of the 9 amino acid, polybasic domain (residues 23–31) is sufficient to dramatically compromise rescuing activity.

### N-terminal deletion mutants do not reverse Tg(F35) pathology

The pathological hallmarks of neurodegeneration in Tg(F35) mice include prominent loss of cerebellar granular neurons (CGNs) and vacuolation of white matter in the cerebellum and other brain areas. In order to determine if the N-terminal deletion mutants were able to rescue these pathological signs, we analyzed brain sections taken from mice co-expressing F35 and either WT, Δ23–31, Δ23–111, or Δ23–134 PrP. Mouse brains were analyzed at 3 weeks (pre-symptomatic), 10 weeks (symptomatic), and time of terminal disease (depending on the different genotypes), and sections were stained with hematoxylin/eosin.

**Figure 5:**
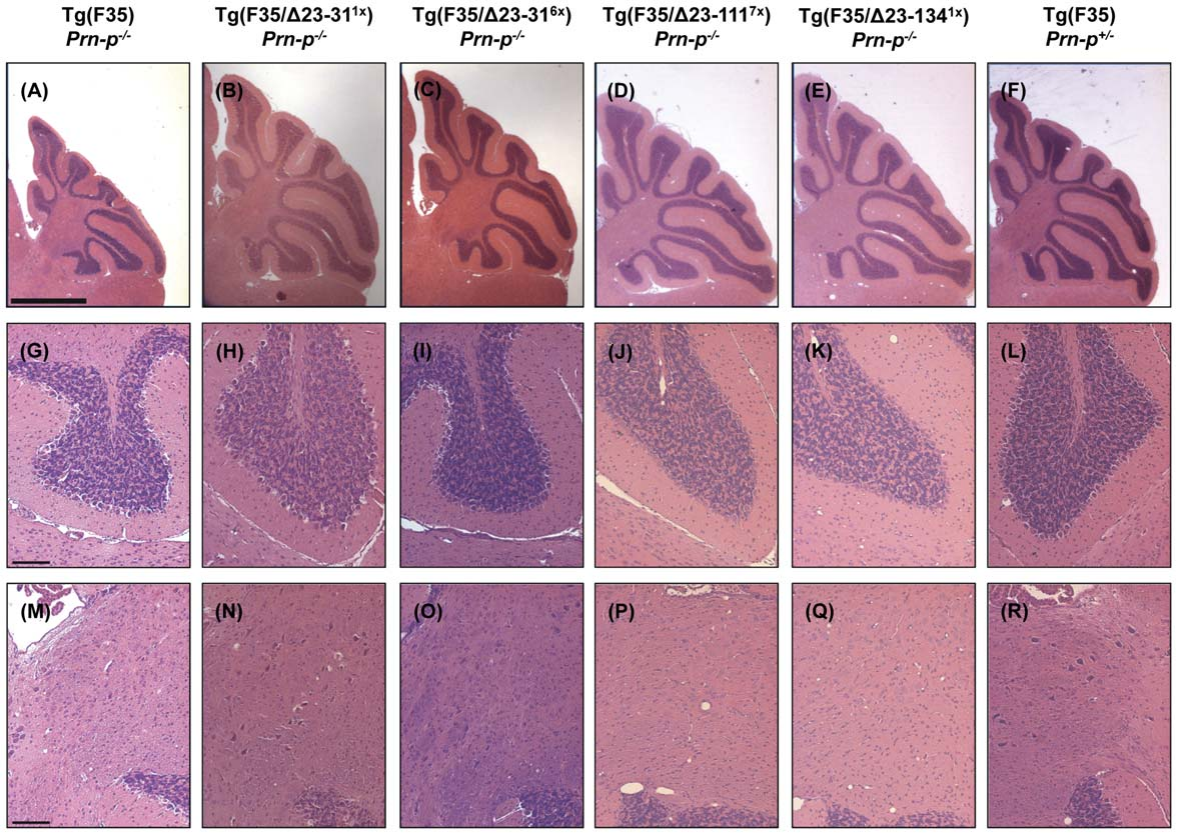
F35 mice co-expressing N-terminal deletion mutants are normal at 3 weeks. Animals of the indicated genotypes were sacrificed at 3 weeks and brain sections were stained with hematoxylin and eosin. Images show the whole cerebellum (A–F), the granule cell layer of the second cerebellar lobe (G–L), and the cerebellar white matter (M–R). Scale bars = 1 mm (A–F) and 100 µm (G–R).

**Figure 6 pone-0025675-g006:**
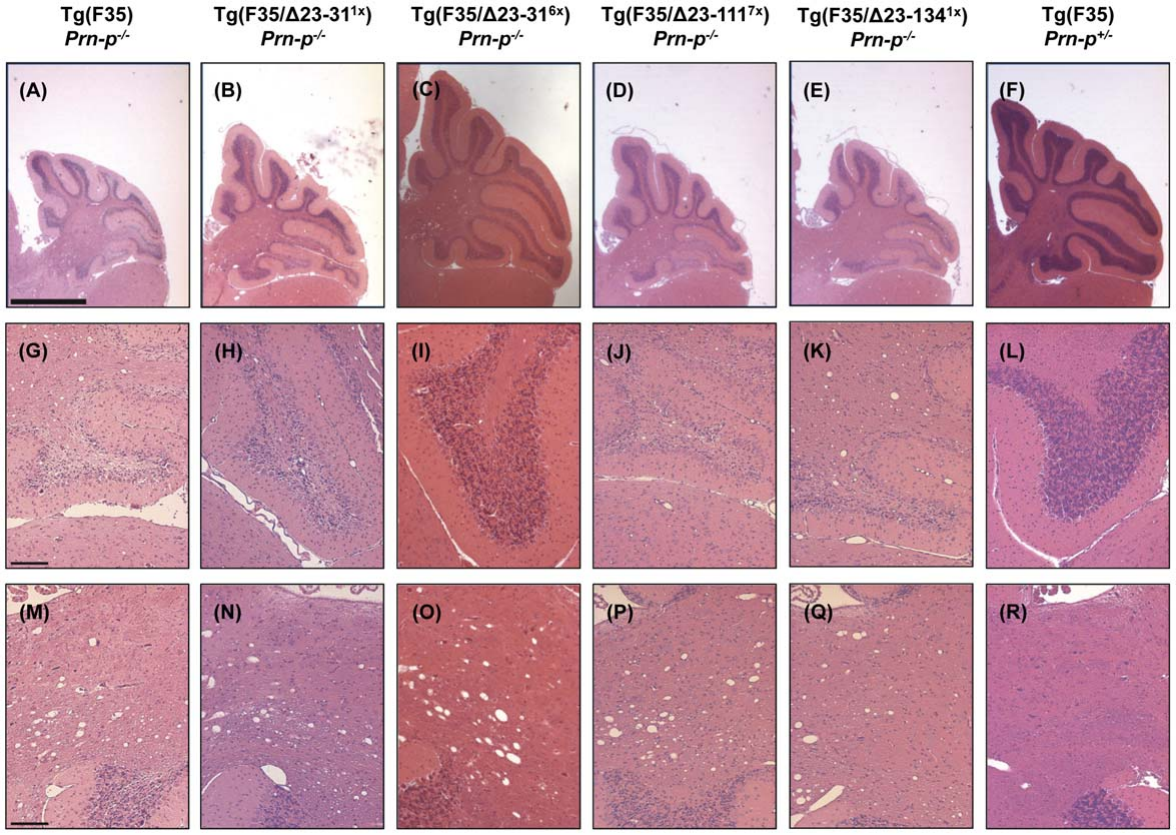
Δ23–31, Δ23–111, and Δ23–134 PrP are impaired in their ability to suppress Tg(F35) neuropathology at 10 weeks. Animals of the indicated genotypes were sacrificed at 10 weeks. Histological staining, order of images, and scale bars are identical to Figure 5. Mice expressing F35 in the presence or absence of Δ23–31^1×^, Δ23–111^7×^, or Δ23–134^1×^ PrP display marked loss of CGNs (G, H, J and K), as well as vacuolation of the cerebellar white matter (M, N, P and Q). Conversely, expression of Δ23–31^6×^ PrP prevents loss of CGNs (I), but not white matter vacuolation (O).

**Figure 7:**
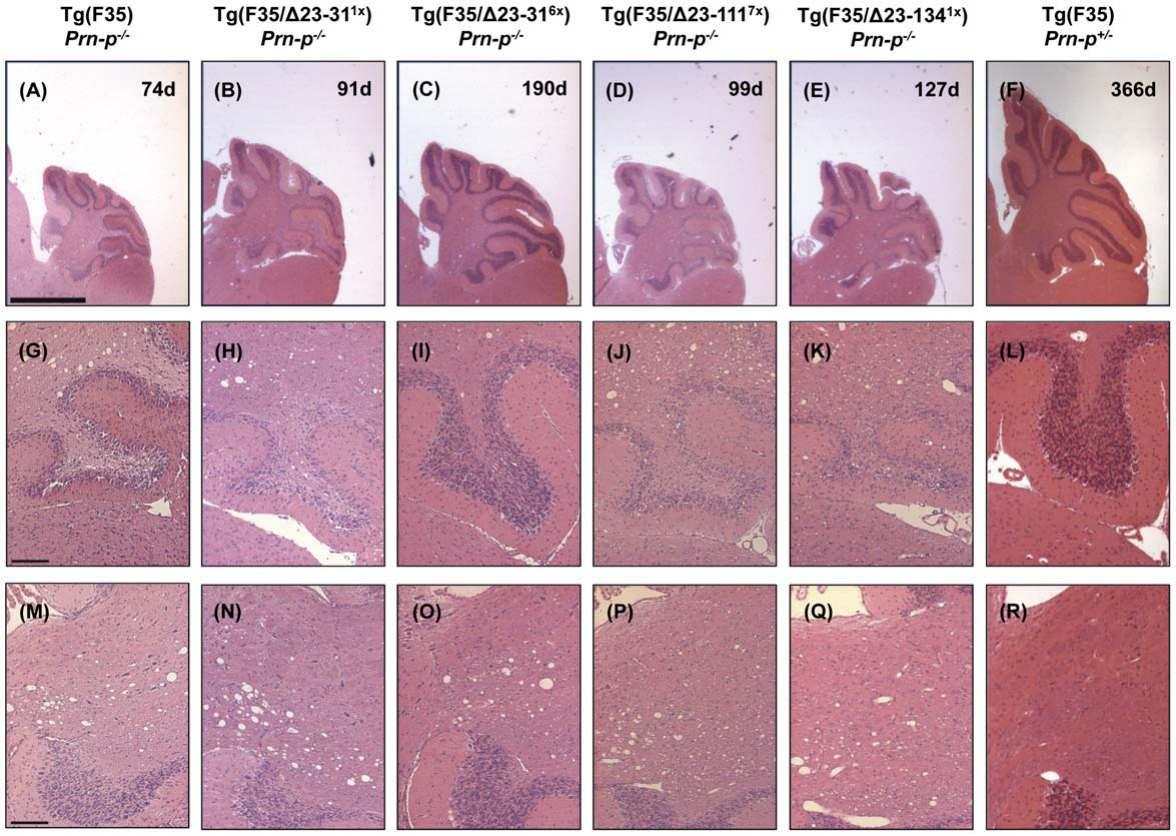
N-terminal deletion mutants do not suppress Tg(F35) neuropathology at time of terminal disease. Histological staining, order of images, and scale bars are identical to Figure 5, except that animals were sacrificed when terminally ill. As for 10 week old animals, Δ23–31^6×^ PrP expression rescues loss of CGNs (I), but not white matter vacuolation (O), while co-expression of Δ23–31^1×^, Δ23–111^7×^, or Δ23–134^1×^ PrP does not prevent either CGN loss (G, H, J and K) or white matter vacuolation (M, N, P and Q).

At 3 weeks, the cerebellum of Tg(F35) mice on a PrP-null background appears slightly shrunken (Figure 5A), although the granule cell layer (Figure 5G) and the white matter (Figure 5M) are still intact. At both 10 weeks and at the time of terminal disease, the cerebellum of these mice is severely atrophic (Figure 6A and 7A), with evident loss of CGNs (Figure 6G and 7G) and white matter vacuolation (Figure 6M and 7M). As expected, no pathological signs were detected at any time point in F35 mice co-expressing 0.5× WT PrP (Figure 5–7, panels F, L and R).

The cerebellum of Tg(F35) mice co-expressing each of the three N-terminal deletion mutants appeared normal at 3 weeks of age (Figure 5 B–E, H–K, N–Q). However, by 10 weeks of age there was noticeable cerebellar atrophy (Figure 6 B, D, E), dramatic loss of CGNs (Figure 6 H, J, K) and accumulation of vacuoles in the cerebellar white matter (Figure 6, N, P, Q) of Tg(F35) mice co-expressing Δ23–31 (1×), Δ23–111 (7×), or Δ23–134 (1×) PrP. These neuropathological changes were even more marked at the time of terminal illness (Figure 7). Unexpectedly, co-expression of Δ23–31 PrP at high levels (6×) prevented the loss of CGNs at both 10 weeks (Figure 6I) and at the time of terminal disease (Figure 7I), although these mice still showed prominent white matter vacuolation at both time points (Figure 6 and 7, O).

These results demonstrate that the N-terminal region of PrP is necessary to fully rescue the pathological changes induced by expression of F35 PrP. The fact that terminally ill Tg(F35/Δ23–31^6×^) mice display prominent white matter vacuolation without substantial granule cell loss suggests that white matter pathology itself is sufficient for causing death in F35 mice.

### F35 does not co-immunoprecipitate with WT or Δ23–31 PrP

One possible explanation for the rescuing ability of WT PrP in Tg(F35) mice is that the normal protein physically interacts with the F35 mutant, preventing its toxic effect. Our results suggest that such interaction would involve residues 23–31. Consequently, deletion of these residues should decrease or abolish binding of WT PrP to F35 PrP.

**Figure 8:**
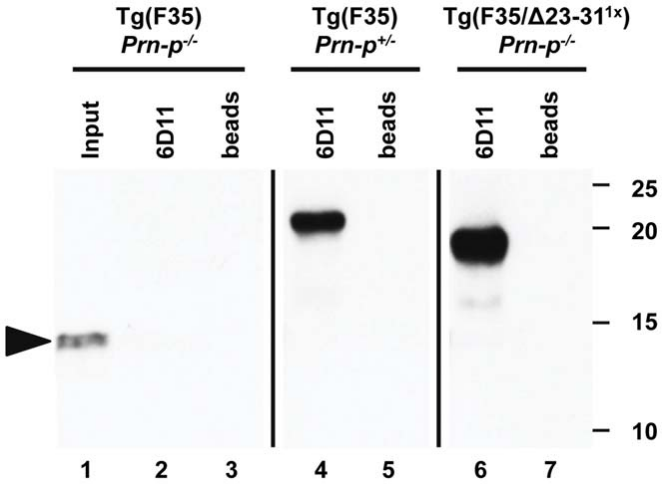
F35 does not interact with either WT PrP or Δ23–31 PrP in co-immunoprecipitation experiments. Brain lysates from mice expressing F35 PrP in the presence or absence of either WT or Δ23–31 PrP were immunoprecipitated with Dynabeads coupled to anti-PrP antibody 6D11, or with naked beads as a control. Immunopreciptated proteins were then analyzed by Western blotting with anti-PrP antibody 6H4. The arrowhead indicates the position of F35 PrP. The faint band appearing in lane 6 between 15 and 20 kDa is distinguishable from the F35 protein, and could represent a C-terminal fragment of Δ23–31 PrP.

To test the possibility that WT but not Δ23–31 PrP directly interacts with the F35 mutant, we performed co-immunoprecipitation experiments on brain homogenates. Beads coated with antibody 6D11, which recognizes an epitope (residues 95–100) deleted in F35, were used to pull-down PrP molecules from F35 mice co-expressing either WT or Δ23–31. After immunoprecipitation, proteins were enzymatically de-glycosylated to discriminate between WT, Δ23–31 and F35 PrPs based on their migration on SDS-PAGE. Antibody 6H4, which recognizes a C-terminal epitope (144–152), was then used to detect all PrP species by Western blotting. As expected, the F35 protein was not immunoprecipitated by naked beads (Figure 8, lane 3), or by 6D11 (Figure 8, lane 2), although the protein was still detected in the input lane (Figure 8, lane 1). Conversely, both WT (Figure 8, lane 4) and Δ23–31 PrP (Figure 8, lane 6) were efficiently immunoprecipitated by 6D11, but not by naked beads (Figure 8, lanes 5 and 7). However, F35 PrP did not co-immunoprecipitate with either WT or Δ23–31 PrP (Figure 8, lanes 4, 6) suggesting that the rescuing activity of WT PrP does not rely on a direct interaction with the toxic mutant.

## Discussion

Expression of WT PrP is known to suppress the spontaneous neurodegenerative phenotype induced by several N-terminal deletion mutants of PrP. For example, Tg(F35) mice expressing Δ32–134 PrP on a *Prn-p* ^−/−^ background become terminally ill within three months after birth, while co-expression of 0.5× endogenous, WT PrP prolongs the lifespan of these animals to more than one year [10]. In the present study, we have defined the regions of PrP participating in this neuroprotective activity, and showed that deletions encompassing the N-terminal polybasic domain (residues 23–31) significantly impair the ability of PrP to reverse neurodegenerative phenotype of Tg(F35) mice. We found that, although Δ23–31 PrP displayed greatly diminished rescuing activity, over-expression of the protein was able to prevent CGN loss, although white matter vacuolation and clinical symptoms still ensued, demonstrating the independent roles of these two kinds of pathology in the death of the animals. We failed to observe co-immunoprecipitation of WT and F35 PrP, suggesting that the rescuing ability of the WT protein does not depend on a physical interaction with the mutant protein. Therefore, deletion of residues 23–31 could compromise PrP neuroprotective activity by disrupting its association with other membrane-bound molecules.

### Residues 23–31 are critical for the neuroprotective activity of PrP

The main conclusion of our study is that N-terminal deletion mutants are significantly impaired in their ability to reverse the phenotype of Tg(F35) mice. This conclusion holds true for three successive deletions including Δ23–31, Δ23–111, and Δ23–134. The most substantial rescue was seen by over-expressing Δ23–31 PrP by six-fold, although even at this expression level the protein did not prevent neurological symptoms or death of Tg(F35) mice. In comparison, an expression level of WT PrP that is 12 times lower (0.5×) is sufficient for fully reversing the Tg(F35) phenotype and allowing the animals to have a normal lifespan. Surprisingly, Δ23–134 PrP, which harbors the longest deletion, showed a mild rescuing effect at physiological expression levels (1×). It is currently unclear why this molecule would display a better rescuing ability than either Δ23–31 or Δ23–111 PrP, when expressed at 1× and 7× respectively. Possibly, the presence of residues between 31 and 134 negatively impacts whatever interactions are important for the rescuing activity of PrP.

While this is the first study to examine the role of the N-terminal, polybasic domain in suppressing the phenotype of Tg(F35) mice, previous studies have investigated whether the N-terminal domain of PrP can exert a neuroprotective activity. A peptide corresponding to PrP residues 23–50 has been shown to reduce the formation of reactive oxygen species in response to serum deprivation in cultured cells [30]. Other studies have analyzed the ability of two N-terminally deleted PrPs (Δ23–88 and Δ25–50) to reverse neurodegeneration in mice ectopically expressing Doppel [18], [31]. Since it lacks the flexible N-terminus, Doppel is structurally similar to the F35 protein, implying that these proteins may induce toxicity via a similar mechanism. Interestingly, while Δ23–88 PrP completely lacked a neuroprotective ability, the expression of Δ25–50 PrP led to a rescue of Doppel-induced neurodegeneration [18], [31], suggesting that residues 23 and 24 by themselves, but not the octapeptide repeats, may impart some protective activity. These data, taken together with those in our study, suggest that the extreme N-terminus of PrP represents a primary determinant of its neuroprotective activity in both Doppel and Tg(F35) mice.

In addition to playing a role in neuroprotection, residues 23–31 also appear to be important in several neurotoxic activities of PrP. For example, mice expressing Δ23–134 PrP, which is equivalent to the F35 mutant missing residues 23–31, showed no evidence of neurodegeneration [29]. Additionally, either deleting or mutating residues 23–31 in the context of Δ105–125 PrP completely abrogates the ion channel activating and aminoglycoside-sensitizing activities of this protein in cells [29], [32]. Collectively, these results demonstrate a critical role for the N-terminal, polybasic domain in regulating both the neurotoxic and neuroprotective functions of PrP.

### White matter pathology and neuronal loss in F35 mice are mechanistically distinct

We have observed that terminally ill Tg(F35) mice over-expressing Δ23–31 PrP by six-fold displayed extensive white matter pathology with little granule cell loss. The details of this white matter pathology have not been dissected, and may be related to either axon or myelin dysfunction. However, the general theme of white matter pathology in the absence of CGN loss is paralleled by several other transgenic models, including Tg(Δ94–134) [11] and Tg(Δ105–125/Tga20) [12]. Moreover, it has been reported that oligodendrocyte-specific expression of WT PrP reversed white matter pathology and dramatically improved survival in Tg(F35) and Tg(Dpl) mice without preventing neuronal loss [33]. Collectively, these results suggest that white matter abnormalities and neuronal loss are mechanistically distinct, and that the former pathology may be the immediate cause of clinical symptoms and death in several kinds of Tg mice expressing toxic PrP mutants or Doppel. Interestingly, recent work has shown that PrP may play a role in myelin maintenance [34], raising the possibility that this functional role may be subverted by certain mutations in the PrP molecule.

### The naturally occurring, C1 proteolytic fragment of PrP is not neuroprotective

The PrP molecule expressed by Tg(Δ23–111) mice is equivalent to a physiologically occurring, C-terminal cleavage fragment of PrP termed C1. C1 is produced by cleavage between residues 111 and 112 by the ADAM10 and ADAM17 proteases [35]. This cleavage leaves the C-terminal half of PrP, composed of residues 112–230, anchored to the plasma membrane, and releases an N-terminal fragment called N1. Previous work has shown that *Prn-p* ^−/−^ mice display a chronic demyelinating polyneuropathy, and that this pathology is rescued by co-expression of transgenes that result in production of C1 but not by transgenes encoding PrP forms non-permissive for cleavage [34]. These authors concluded that regulated proteolysis of PrP^C^ is essential for myelin maintenance. In contrast, our data suggest that C1 (Δ23–111) is incapable of preventing the neuropathological changes, including white matter pathology, induced by F35 PrP. While it is possible that Tg(F35) and *Prn-p* ^−/−^ mice suffer from different types of white matter dysfunction, an alternative hypothesis is that N1, rather than C1, is necessary for the rescue effect in both kinds of mice. The lines examined by Bremer et al. that are non-permissive for cleavage would generate neither N1 nor C1, while our Tg(Δ23–111) lines produce only a C1-like fragment. This explanation is supported by previous experiments showing that N1 has a neuroprotective function in retinal cells via modulation of the p53 pathway both *in vitro* and *in vivo* [36]. Although more work remains to elucidate the significance of the N1/C1 cleavage in the brain, we have shown that the C1 protein is incapable of providing a neuroprotective effect in the context of F35-induced neurodegeneration.

### How do residues 23–31 play a role in the neuroprotective activity of PrP?

One explanation is that these residues form part of a binding site between PrP and an interacting molecule on the cell surface. In this study, we provided evidence that WT and F35 PrP do not physically interact, although it remains possible that these two proteins engage in a weak or transient interaction that is not detectable in the co-immunoprecipitation experiment we performed. Previous work suggested that PrP is cabable of forming a dimer [37], [38], [39], but the results of our co-immunoprecipitation experiment indicate that such dimerization may not occur between heterologous molecules of PrP, such as F35 and WT, at least under the conditions we have used.

Our results raise the possibility that WT rescuing activity relies on interaction with an alternative binding partner whose binding to PrP is dependent on the presence of residues 23–31. Previous studies have identified molecules (including proteins, glycans, and lipids) that interact with PrP, some of which have been found to bind specifically to the N-terminus of PrP. These include the low-density lipoprotein receptor-related protein 1 (LRP1), which modulates the endocytosis of PrP [40]. Disruption of this region prevents this endocytosis of PrP [19], [20], [21], [41], and influences its half-life and rate of trafficking to the plasma membrane [41]. These residues are also a binding site for GAGs [22], [23], [24], which can mediate binding between PrP and the 37 kDa/67 kDa laminin receptor [42]. Additionally, the polybasic region is capable of interacting with the plasma membrane as a protein transduction domain [25] or an antimicrobial peptide [43], although several studies indicate that its ability to insert into the membrane also requires the presence of the octapeptide repeat region [44], [45]. In PrP that is targeted to the cytoplasm due to abnormal folding or processing, these residues can function as a nuclear localization signal [46] and interact with tubulin [47], although these interactions may not be physiologically relevant in the presence of normally processed PrP, which is localized primarily to the outer leaflet of the plasma membrane. Whether these or other, undefined interactions are relevant to the neuroprotective function of PrP remains unresolved.

Of note, a recent report identifies residues 23–27 of PrP as one of the two sites that bind oligomers of the Alzheimer's Aβ peptide [48], suggesting a role for this region in mediating the synaptotoxic effects of these oligomers. Given the role of the N-terminal polybasic domain in determining the neuroprotective properties of PrP, as well as its binding to other toxic oligomers [49], this region may prove to be an important therapeutic target in prion as well as other neurodegenerative disorders.
